# Efficacy, duration of protection, birth outcomes, and infant growth associated with influenza vaccination in pregnancy: a pooled analysis of three randomised controlled trials

**DOI:** 10.1016/S2213-2600(19)30479-5

**Published:** 2020-06

**Authors:** Saad B Omer, Dayna R Clark, Shabir A Madhi, Milagritos D Tapia, Marta C Nunes, Clare L Cutland, Eric A F Simões, Anushka R Aqil, Joanne Katz, James M Tielsch, Mark C Steinhoff, Niteen Wairagkar, BMGF Supported Maternal Influenza Immunization Trials Investigators Group, William Blackwelder, Joseph Bresee, Flanon Coulibaly, Boubacar Diallo, Fatoumata Diallo, Wilbur Chen, Moussa Doumbia, Fadima Cheick Haidara, Adama Mamby Keita, Alexander Klimov, Mamoudou Kodio, Karen Kotloff, Myron M. Levine, Vladimir Mishcherkin, Uma Onwuchekwa, Sandra Panchalingam, Marcela Pasetti, Doh Sanogo, Samba Sow, Milagritos Tapia, Boubou Tamboura, Ibrahim Teguete, Sharon Tennant, Awa Traore, John Treanor, Janet A. Englund, Joanne Katz, Subarna K. Khatry, Jane Kuypers, Steven C. LeClerq, Luke C. Mullany, Laxman Shrestha, Mark C. Steinhoff, James M. Tielsch, Peter V. Adrian, Clare L. Cutland, Andrea Hugo, Stephanie Jones, Locadiah Kuwanda, Keith P. Klugman, Shabir A. Madhi, Kathleen M. Neuzil, Nadia van Niekerk, Marta C. Nunes, Justin R. Ortiz, Eric A.F. Simões, Florette Treurnicht, Marietjie Venter, Avy Violari, Adriana Weinberg

**Affiliations:** a Yale Institute for Global Health, New Haven, CT, USA; b Department of Internal Medicine (Infectious Diseases), Yale School of Medicine, New Haven, CT, USA; c Yale School of Nursing, New Haven, CT, USA; d Department of Epidemiology of Microbial Diseases, Yale School of Public Health, New Haven, CT, USA; e Department of Epidemiology, Emory University Rollins School of Public Health, Atlanta, GA, USA; f Medical Research Council: Respiratory and Meningeal Pathogens Research Unit, Faculty of Health Sciences, University of the Witwatersrand, Johannesburg, South Africa; g Department of Science and Technology/National Research Foundation, Faculty of Health Sciences, University of the Witwatersrand, Johannesburg, South Africa; h Centre pour le Développement des Vaccins, Bamako, Mali; i Center for Vaccine Development, University of Maryland School of Medicine, Baltimore, MD, USA; j Section of Infectious Diseases, Department of Pediatrics, University of Colorado School of Medicine and Children's Hospital Colorado, Aurora, CO, USA; k Department of Epidemiology, Center for Global Health Colorado School of Public Health, Aurora, CO, USA; l Department of Health, Behavior, Society, Johns Hopkins University Bloomberg School of Public Health, Baltimore, MD, USA; m Department of International Health, Johns Hopkins University Bloomberg School of Public Health, Baltimore, MD, USA; n Department of Global Health, Milken Institute School of Public Health, George Washington University, Washington, DC, USA; o Cincinnati Children's Hospital Global Health Center, Cincinnati, OH, USA; p Bill & Melinda Gates Foundation, Seattle, WA, USA; q Vaccines For All, Pune, India

## Abstract

**Background:**

Maternal influenza immunisation can reduce morbidity and mortality associated with influenza infection in pregnant women and young infants. We aimed to determine the vaccine efficacy of maternal influenza immunisation against maternal and infant PCR-confirmed influenza, duration of protection, and the effect of gestational age at vaccination on vaccine efficacy, birth outcomes, and infant growth up to 6 months of age.

**Methods:**

We did a pooled analysis of three randomised controlled trials done in Nepal (2011–2014), Mali (2011–2014), and South Africa (2011–2013). Pregnant women, gestational age 17–34 weeks in Nepal, 28 weeks or more in Mali, and 20–36 weeks in South Africa, were enrolled. Women were randomly assigned 1:1 to a study group, in which they received trivalent inactivated influenza vaccine (IIV) in all three trials, or a control group, in which they received saline placebo in Nepal and South Africa or quadrivalent meningococcal conjugate vaccine in Mali. Enrolment at all sites was complete by April 24, 2013. Infants and women were assessed for respiratory illness, and samples from those that met the case definition were tested for influenza by PCR testing. Growth measurements, including length and weight, were obtained at birth at all sites, at 24 weeks in South Africa, and at 6 months in Nepal and Mali. The three trials are registered with ClinicalTrials.gov, numbers NCT01430689, NCT01034254, and NCT02465190.

**Findings:**

10 002 women and 9800 liveborn infants were included. Pooled efficacy of maternal vaccination to prevent infant PCR-confirmed influenza up to 6 months of age was 35% (95% CI 19 to 47). The pooled estimate was 56% (28 to 73) within the first 2 months of life, 39% (11 to 58) between 2 and 4 months, and 19% (–9 to 40) between 4 and 6 months. In women, from enrolment during pregnancy to the end of follow-up at 6 months postpartum, the vaccine was 50% (95% CI 32–63) efficacious against PCR-confirmed influenza. Efficacy was 42% (12 to 61) during pregnancy and 60% (36 to 75) postpartum. In women vaccinated before 29 weeks gestational age, the estimated efficacy was 30% (–2 to 52), and in women vaccinated at or after 29 weeks, efficacy was 71% (50 to 83). Efficacy was similar in infants born to mothers vaccinated before or after 29 weeks gestation (34% [95% CI 12 to 51] *vs* 35% [11 to 52]). There was no overall association between maternal vaccination and low birthweight, stillbirth, preterm birth, and small for gestational age. At 6 months of age, the intervention and control groups were similar in terms of underweight (weight-for-age), stunted (length-for-age), and wasted (weight-for-length). Median centile change from birth to 6 months of age was similar between the intervention and the control groups for both weight and length.

**Interpretation:**

The assessment of efficacy for women vaccinated before 29 weeks gestational age might have been underpowered, because the point estimate suggests that there might be efficacy despite wide CIs. Estimates of efficacy against PCR-confirmed influenza and safety in terms of adverse birth outcomes should be incorporated into any further consideration of maternal influenza immunisation recommendations.

**Funding:**

Bill & Melinda Gates Foundation.

## Introduction

Pregnant women and young infants have disproportionately high morbidity and mortality associated with influenza.^1^, ^2^, ^3^ Immunisation in pregnancy can protect both the woman and her young infant. Given that no vaccines are available for infants younger than 6 months of age, maternal vaccination during pregnancy can potentially contribute to reduction of global neonatal and infant morbidity and mortality associated with influenza.

A small randomised controlled trial (RCT) done in Bangladesh reported a lower incidence of rapid-test-confirmed influenza among infants of women who received inactivated influenza vaccine (IIV) in pregnancy than was noted in infants of mothers who did not receive IIV in pregnancy.^4^ Although this trial was the first to show protection of infants against laboratory-confirmed influenza after maternal immunisation, it had several limitations. Namely, enzyme-based rapid influenza tests—widely used when the trial was done—have relatively modest sensitivity and specificity.^5^, ^6^, ^7^ Moreover, the trial had a relatively small sample size with low statistical power for stratified analyses (eg, vaccine efficacy by infant age and gestational age at vaccination). Given substantial heterogeneity in influenza burden and epidemiology by location and over time, generalisability of the Bangladesh trial was also limited.

**Research in context**

**Evidence before this study**

We searched PubMed for randomised controlled trials published between Jan 1, 2008, and Dec 6, 2019, and included the terms “maternal influenza immunisation” or “maternal influenza vaccination”. This search yielded 47 results, including reports of four trials on maternal influenza immunisation in low-income and middle-income countries that have shown efficacy against influenza infection in infants, with estimates ranging from 30% to 63%. The first trial, although it showed efficacy against infant influenza, had several limitations. The sensitivity and specificity of the rapid influenza test used at the time the trial was done was modest, and the sample size was too small for stratified analyses on infant age or gestational age at vaccination. The other three trials, used in this pooled analysis, were planned to address these limitations, and presented efficacy estimates for maternal influenza immunisation against maternal and infant PCR-confirmed influenza and age-specific estimates using non-uniform age-groups. Regarding protection against adverse birth outcomes such as low birthweight, the four trials presented mixed evidence.

**Added value of this study**

Given the findings reported by individual trials and because maternal influenza immunisation is standard of care in many countries, it will be difficult to justify placebo-controlled randomised controlled trials in the future. Therefore, the available data should be evaluated extensively. This pooled analysis, planned before the completion of the three randomised controlled trials, has adequate power for additional analyses not reported in the individual trial reports. We report overall vaccine efficacy of maternal influenza immunisation against maternal and infant PCR-confirmed influenza, duration of infant protection, the effect of gestational age at vaccination on vaccine efficacy, and the effect of vaccination on adverse birth outcomes and infant growth.

**Implications of all the available evidence**

Global and several national recommendations to provide influenza immunisation to pregnant women are supported by our findings. When considering future recommendations of maternal influenza immunisation, efficacy estimates and evidence of impact against PCR-confirmed influenza and safety in terms of adverse birth outcomes should be incorporated.

To strengthen the evidence base for maternal influenza immunisation and address the limitations of the Bangladesh trial, the Bill & Melinda Gates Foundation sponsored three RCTs of maternal influenza immunisation in Nepal, Mali, and South Africa.^5^, ^6^, ^7^, ^8^ Previous reports of these trials included analyses of site-specific vaccine efficacy and reported infant age-specific efficacy using non-uniform age-groups, including less than 1, 2, 3, 4, 5, and 6 months of age in Mali and less than 8, 16, and 24 weeks of age in South Africa.^9^, ^10^ Moreover, efficacy against infant outcomes was reported by gestational age at the time of maternal vaccine administration in Nepal, although not in Mali or South Africa.^11^

There is also conflicting evidence on whether influenza infection during pregnancy is associated with adverse birth outcomes, and evidence of protection against adverse birth outcomes, such as low birthweight, was mixed between the four RCTs of maternal influenza immunisation.^8^, ^9^, ^12^, ^13^, ^14^, ^15^, ^16^, ^17^ Although the trial done in Bangladesh reported a protective effect on birthweight, as did the trial in Nepal when combining data for two cohorts, this association was not observed in South Africa or Mali.^8^, ^9^, ^12^, ^17^ It is also necessary to evaluate the effect of maternal influenza immunisation on birth outcomes from a safety perspective. Additionally, the effect of maternal influenza immunisation on respiratory illness in infants could plausibly have consequences for infant growth.

In this pooled analysis, we aimed to assess the overall vaccine efficacy of maternal influenza immunisation against maternal and infant PCR-confirmed influenza, duration of protection, the effect of gestational age at vaccination on vaccine efficacy, adverse birth outcomes, and infant growth up to 6 months of age.

## Methods

### Study design and participants

Previous publications have described each of the three clinical trials.^8^, ^9^, ^12^ Funded by the Gates Foundation, the trials were initially designed as separate studies with overlapping features. Trial procedures were then coordinated for future pooled analyses from the planning phase onward, before completion of the trials, as previously outlined.^5^

Pregnant women were enrolled from April 25, 2011, to April 24, 2013, in Nepal, Sep 12, 2011, to April 18, 2013, in Mali, and March 3, 2011, to July 2, 2012, in South Africa. Infant follow-up visits ended on April 13, 2014, in Nepal, Jan 28, 2014, in Mali, and May 20, 2013, in South Africa. Infants were followed up for 6 months in Nepal and Mali and 24 weeks in South Africa. The studies in Nepal and Mali enrolled and vaccinated women year round, and the study in South Africa coincided enrolment with the influenza season. Multiple peaks of influenza activity were observed in Nepal and two peaks a year were observed in Mali (February and September–October). In South Africa, there were two peaks in the 2011 season (June and September) and one peak in 2012 (August).

Pregnant women were screened and enrolled from nine Village Development Committees in rural southern Nepal (vaccinated at 17–34 weeks gestational age). Women accessing prenatal care were screened and enrolled in Bamako, Mali (vaccinated at ≥28 weeks gestational age) and Soweto, South Africa (vaccinated at 20–36 weeks gestational age). The overall median gestational age at vaccination, 29 weeks, was used in the analysis to stratify early and late gestational age at vaccination. In Nepal, the dates of last menstrual period were prospectively collected to calculate gestational age, although the studies in Mali and South Africa both used estimates at vaccination. In Mali and South Africa, ultrasound, uterine height, and date of last menstrual period were used.

Verbal informed consent was obtained in Nepal, and written informed consent was obtained in South Africa. All women provided informed consent in Mali. If a participant in Mali was illiterate, consent was obtained in the presence of a literate witness after listening to an audio recording of the consent form. The study protocols were reviewed and approved by institutional review boards of partner entities: Emory University; University of Maryland; the Ministry of Health, Mali; Johns Hopkins Bloomberg School of Public Health; the Institute of Medicine at Tribhuvan University, Kathmandu, Nepal; Nepal Health Research Council, Kathmandu, Nepal; and University of Witwatersrand, Johannesburg.^5^, ^8^, ^9^, ^12^ The Emory University institutional review board performed ongoing and periodic review of the pooled analyses.

### Randomisation and masking

Women were randomly assigned 1:1 to a study group, in which they received trivalent IIV (VAXIGRIP; Sanofi-Pasteur, sourced from Mumbai, India in Nepal, and from Lyon, France in Mali and South Africa) in all three trials, or a control group, in which they received saline placebo in Nepal and South Africa or quadrivalent meningococcal conjugate vaccine (Menactra; Sanofi Pasteur, Lyon, France) in Mali. In Nepal, women were assigned in blocks of eight, stratified by gestational age at enrolment (17–25 weeks *vs* 26–34 weeks), using sealed envelopes with the participant number on the outside and an allocation code within. In Mali, women were assigned using a computer-generated list with blocks of six or 12. In South Africa, randomisation was also computer-generated in blocks of 30 by enrolment site.

### Procedures

Women were immunised with a single 0·5 mL dose of either IIV or control product injected into the deltoid muscle. Infants and women at all sites were assessed weekly for illness through active surveillance, and samples were tested for influenza by PCR, as were samples from participants hospitalised with respiratory illness. At all three sites, the influenza strains were determined, including H1N1 and H3N2 influenza A strains or influenza B. Vaccine and non-vaccine matched strains were included in analyses. Across sites, the most predominant influenza strain was influenza H3N2 in infants (37·0 cases per 1000 person-years among controls), and influenza B in women (20·0 cases per 1000 person-years among controls). With the exception of strain-specific estimates, we included all influenza strains in pooled vaccine efficacy estimates.

Infant growth measurements, including length and weight, were obtained at birth at all three sites, at 24 weeks in South Africa, and 6 months in Nepal and Mali. Measurements were excluded if infants were older than 72 h when measured at birth. Infants were also excluded if they were younger than 150 days or 210 days or older at their last visit.

### Outcomes

Outcomes analysed in this pooled analysis include overall vaccine efficacy of maternal influenza immunisation against maternal and infant PCR-confirmed influenza, duration of maternal and infant protection, the effect of gestational age at vaccination on vaccine efficacy, adverse birth outcomes (low birthweight, stillbirth, preterm birth, and small for gestational age), and infant growth up to 6 months of age (infant weight-for-age, weight-for-length, length-for-age, median centile change from birth to 6 months, and mean weight and length at birth at 6 months).

### Statistical analysis

A combined cohort size of 10 000 provided more than 99% power to see a 35% difference in PCR-confirmed infant influenza infection based on a baseline incidence of approximately 0·200 cases per infant-year (assuming 0·500 person-years per infant), more than 99% power to detect a 20% difference in PCR-confirmed maternal influenza infection based on a baseline incidence of around 0·040 cases per person-year (assuming 0·500 person-years per mother), 80% power to detect a 40% difference in stillbirth based on a baseline incidence of around 0·015 cases per woman, and 80% power to detect a 18% difference in preterm birth based on a baseline incidence of 0·120 cases per live-birth. The combined analysis also had 80% power to detect a 15% difference in low birthweight with a combined cohort size of 9000 and a baseline incidence of around 0·150 cases per infant, and 80% power to detect a 13% difference in small for gestational age with a combined cohort size of 8500 and a baseline incidence of 0·300 cases per infant.

For infant growth analyses, infant weight-for-age, weight-for-length, and length-for-age Z scores and percentiles were determined using the WHO growth standards.^18^ Analyses included cutoffs for underweight (weight-for-age Z score <–2), severely underweight (weight-for-age Z scores <–3), wasting (weight-for-length Z score <–2), severe wasting (weight-for-length Z scores <–3), stunting (length-for-age Z score <–2), and severe stunting (length-for-age Z scores <–3). Weight-for-age, weight-for-length, and length-for-age Z scores greater than 7 or less than negative 7 were excluded, resulting in excluding 50 infants from the at-birth analysis, and 17 infants from the 6-month analysis. Intergrowth newborn size standards were used to identify small for gestational age infants.^19^ Birthweight less than 2500 g was considered low birthweight.

Data was pooled and analysed using a one-stage meta-analysis. Poisson regression models were used to calculate incidence rate ratios (IRR), and pooled Poisson models were based on random intercept models. Goodness-of-fit χ^2^ tests were used to assess the fit of the Poisson models. Log-binomial regressions were used to estimate risk ratios (RRs), and robust CIs were used. Pooled models were adjusted for the effects of site (each of the three trial locations), and interaction by site was evaluated for each pooled analysis. Interaction by site was significant for efficacy of maternal vaccination against PCR-confirmed influenza in infants less than 4 months of age (p=0·04), during the full study period for women (p=0·03), less than 4 months after vaccination for women (p=0·004), less than 6 months after vaccination for women (p=0·005), efficacy against H1N1 for women (p=0·01), and the low birthweight analysis (p=0·01). To further assess heterogeneity across sites by *I*^2^ testing, we did two-stage meta-analyses with site RRs. Vaccine efficacy was calculated as: (1 – IRR) × 100. Changes from birth to 6 months in median weight and length percentiles between intervention and control groups were compared using Wilcoxon Rank Sum Test. Mean weight and length at birth and 6 months were compared across study groups using two-sample t tests.

Infants and women were considered always at risk for influenza infection and were censored at 180 days postpartum for Nepal and Mali and at 175 days postpartum in South Africa as per protocol. In age group analyses, 61 days was considered 2 months of age, and 122 days was considered 4 months of age. Analyses were as consistent as possible across sites, in terms of case definitions, person-time calculations, and data cleaning, for example. Therefore, numbers from this paper might differ slightly from site-specific publications. Women were excluded from during and after pregnancy analyses if the date of delivery was unknown, because we were not able to determine if the influenza episode occurred during or after pregnancy. Statistical analyses were done using Stata version 14.2.

The three trials were registered with ClinicalTrials.gov, numbers NCT01430689, NCT01034254, and NCT02465190.

### Role of the funding source

The funder of the study had no role in study design or conduct of the three studies and no role in the data collection for the pooled analysis, but provided feedback on the pooled analysis study design, data analysis, data interpretation, and writing of the manuscript. The working group of authors had full access to all the data in the study and were responsible for the final decision to submit for publication.

## Results

**Figure 1:**
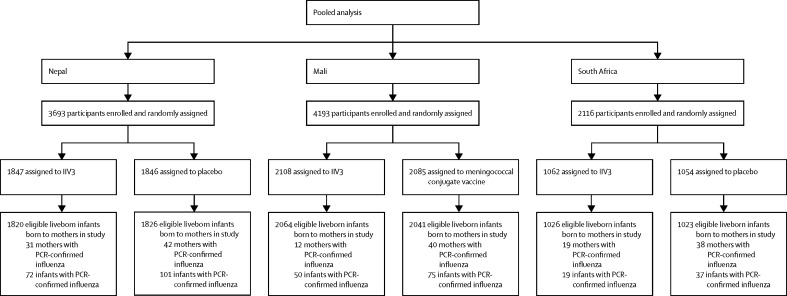
Study profile *Numbers do not sum because not every assigned mother had a liveborn infant, and some had twins.

Across all three sites, 10 002 women (5017 assigned to IIV and 4985 assigned to control) gave birth to 9800 total liveborn eligible infants (4910 livebirths to women who received IIV and 4890 livebirths to women who received control). Miscarriages, abortions, and women lost to follow up before delivery were excluded from analyses. Stillbirths were also excluded with the exception of the stillbirth analysis (figure 1). Previous publications^8^, ^9^, ^12^ have described the distribution of infant and maternal characteristics, and the two study groups were similar in terms of maternal age and gestational age at enrolment.

The pooled vaccine efficacy of maternal IIV to prevent infant PCR-confirmed influenza among infants until 6 months of age was 35% (95% CI 19 to 47; table 1). Within the first 2 months of infant life, the pooled efficacy of maternal vaccination was 56% (28 to 73); and the efficacy was 46% (28 to 60) in the first 4 months of life. Pooled vaccine efficacy estimates in infants only accounting for the first episode of influenza were similar to the analyses accounting for all episodes of influenza (table 1; appendix p 2). When stratified by site, maternal vaccination was efficacious at all three sites in protecting infants against PCR-confirmed influenza (table 1). In the analysis of vaccine efficacy with non-cumulative age intervals, efficacy was 39% (11 to 58) between 2 and 4 months of age and not significant between 4 and 6 months of age (vaccine efficacy 19%; 95% CI −9 to 40; table 1).

**Table 1:** Efficacy of maternal inactivated influenza vaccine on episodes of PCR-confirmed influenza in infants born to women vaccinated at any time prepartum

		**Intervention**			**Control**			**Incidence rate ratio (95% CI)**	**Vaccine efficacy, % (95% CI)**	**p value**
		Cases	Person-time*	Incidence, per 1000 infant-years	Cases	Person-time*	Incidence, per 1000 infant-years			
**Cumulative episodes**										
≤2 months of age										
	Nepal	20	303·4	65·9	31	304·4	101·8	0·65 (0·37 to 1·14)	35% (−14 to 63)	0·13
	Mali	2	332·3	6·0	10	329·9	30·3	0·20 (0·04 to 0·91)	80% (19 to 96)	0·04
	South Africa	2	168·3	11·9	13	167·8	77·5	0·15 (0·03 to 0·68)	85% (32 to 97)	0·01
	Pooled	24	804·0	29·8	54	802·1	67·3	0·44 (0·27 to 0·72)	56% (28 to 73)	0·001
≤4 months of age										
	Nepal	43	606·7	70·9	61	608·7	100·2	0·71 (0·48 to 1·04)	29% (−4 to 52)	0·08
	Mali	11	651·2	16·9	35	649·4	53·9	0·31 (0·16 to 0·62)	69% (38 to 84)	0·001
	South Africa	14	334·2	41·9	30	333·3	90·0	0·46 (0·25 to 0·87)	54% (13 to 75)	0·02
	Pooled	68	1592·1	42·7	126	1591·3	79·2	0·54 (0·40 to 0·72)	46% (28 to 60)	<0·001
≤6 months of age										
	Nepal	74	896·9	82·5	105	899·9	116·7	0·71 (0·52 to 0·95)	29% (5 to 48)	0·02
	Mali	50	931·0	53·7	77	929·0	82·9	0·65 (0·45 to 0·92)	35% (8 to 55)	0·02
	South Africa	19	468·6	40·6	37	466·8	79·3	0·51 (0·30 to 0·88)	49% (12 to 70)	0·02
	Pooled	143	2296·4	62·3	219	2295·7	95·4	0·65 (0·53 to 0·81)	35% (19 to 47)	<0·001
**Non-cumulative episodes**										
≤2 months of age										
	Nepal	20	303·4	65·9	31	304·4	101·8	0·65 (0·37 to 1·14)	35 (−14 to 63)	0·13
	Mali	2	332·3	6·0	10	329·9	30·3	0·20 (0·04 to 0·91)	80 (19 to 96)	0·04
	South Africa	2	168·3	11·9	13	167·8	77·5	0·15 (0·03 to 0·68)	85 (32 to 97)	0·01
	Pooled	24	804·0	29·8	54	802·1	67·3	0·44 (0·27 to 0·72)	56 (28 to 73)	0·001
>2–4 months of age										
	Nepal	23	303·3	75·8	30	304·3	98·6	0·77 (0·45 to 1·32)	23 (−32 to 55)	0·34
	Mali	9	318·9	28·2	25	319·4	78·3	0·36 (0·17 to 0·77)	64 (23 to 83)	0·01
	South Africa	12	165·9	72·3	17	165·4	10·3	0·70 (0·34 to 1·47)	30 (−47 to 66)	0·35
	Pooled	44	788·1	55·8	72	789·2	91·2	0·61 (0·42 to 0·89)	39 (11 to 58)	0·01
>4–6 months of age										
	Nepal	31	290·2	106·8	44	291·2	151·1	0·71 (0·45 to 1·12)	29 (−12 to 55)	0·14
	Mali	39	279·7	13·9	42	279·6	15·0	0·93 (0·60 to 1·44)	7 (−44 to 40)	0·74
	South Africa	5	134·3	37·2	7	133·5	52·4	0·71 (0·22 to 2·24)	29 (−124 to 78)	0·56
	Pooled	75	704·3	106·5	93	704·4	132·0	0·81 (0·60 to 1·09)	19 (−9 to 40)	0·17

Infants might have had repeat episodes of influenza.

* Person-time was calculated consistently across sites and might be different from original site publication.

From enrolment during pregnancy to the end of follow-up at 6 months postpartum, the vaccine was 50% (95% CI 32–63) efficacious against PCR-confirmed influenza in women (table 2).

**Table 2:** Efficacy of maternal inactivated influenza vaccine on episodes of PCR-confirmed influenza in women vaccinated at any time prepartum

	**Intervention**			**Control**			**Incidence rate ratio (95% CI)**	**Vaccine efficacy, % (95% CI)**	**p value**
	Cases	Person-time*	Incidence, per 1000 person-years	Cases	Person-time*	Incidence, per 1000 person-years			
**During pregnancy**†									
Nepal	19	563·6	33·7	24	558·7	43·0	0·78 (0·43 to 1·43)	22 (−43 to 57)	0·43
Mali	6	313·7	19·1	18	312·2	57·7	0·33 (0·13 to 0·84)	67 (16 to 87)	0·02
South Africa	11	226·9	48·5	19	223·4	85·0	0·57 (0·27 to 1·20)	43 (−20 to 73)	0·14
Pooled	36	1104·2	32·6	61	1094·3	55·7	0·58 (0·39 to 0·88)	42 (12 to 61)	0·01
**After pregnancy**†									
Nepal	12	910·2	13·2	20	909·7	22·0	0·60 (0·29 to 1·23)	40 (−23 to 71)	0·16
Mali	7	973·5	7·2	23	966·3	23·8	0·30 (0·13 to 0·70)	70 (30 to 87)	0·01
South Africa	6	491·2	12·2	19	488·2	38·9	0·31 (0·12 to 0·78)	69 (13 to 88)	0·01
Pooled	25	2374·9	10·5	62	2364·3	26·2	0·40 (0·25 to 0·64)	60 (36 to 75)	<0·001
**During full study period**									
Nepal	31	1471·3	21·1	44	1466·4	30·0	0·70 (0·44 to 1·11)	30 (−12 to 56)	0·13
Mali	12	1259·6	9·5	41	1253·8	32·7	0·29 (0·15 to 0·55)	71 (45 to 85)	<0·001
South Africa	19	707·4	26·8	38	700·8	54·2	0·50 (0·28 to 0·86)	50 (14 to 72)	0·01
Pooled	62	3438·3	18·0	123	3421·0	36·0	0·50 (0·37 to 0·68)	50 (32 to 63)	<0·001

Women might have had repeat episodes of influenza.

* Person-time was calculated consistently across sites and might be different from original site publication.

† Women without a date of delivery were excluded from the analyses.

In the analysis restricted to influenza episodes in pregnancy, the vaccine efficacy was 42% (12–61) and in the postpartum period it was 60% (36–75; table 2).

2 months following vaccination, the overall vaccine efficacy was 49% (95% CI 16 to 69) among women. 4 months after vaccination, there was 44% pooled efficacy (18 to 61); although the vaccine was effective in Mali during this period (vaccine efficacy 73%; 95% CI 39 to 88), this was not true of the other sites. There was 49% pooled efficacy (95% CI 29 to 63) 6 months after vaccination (table 3). During this period, the vaccine was not effective in Nepal (vaccine efficacy 13%, 95% CI −46 to 49), although it was in Mali (76%, 50 to 88) and South Africa (50%, 13 to 72; table 3). During the non-cumulative period 2–4 months after vaccination, the vaccine efficacy was statistically non-significant (36%, −12 to 64), and during the period 4–6 months after vaccination, efficacy was 63% (95% CI 24 to 82; table 3).

**Table 3:** Efficacy of maternal inactivated influenza vaccine on episodes of PCR-confirmed influenza in women vaccinated at any time prepartum

		**Intervention**			**Control**			**Incidence rate ratio (95% CI)**	**Vaccine efficacy, % (95% CI)**	**p value**
		Cases	Person-time*	Incidence, per 1000 person-years	Cases	Person-time*	Incidence, per 1000 person-years			
**Cumulative episodes**										
≤2 months after vaccination										
	Nepal	9	307·8	29·2	12	307·7	39·0	0·75 (0·32 to 1·78)	25 (−78 to 68)	0·51
	Mali	6	350·1	17·1	19	346·7	54·8	0·31 (0·12 to 0·79)	69 (21 to 88)	0·01
	South Africa	8	175·8	45·5	14	173·5	80·7	0·56 (0·24 to 1·34)	44 (−34 to 76)	0·20
	Pooled	23	833·7	27·6	45	827·9	54·4	0·51 (0·31 to 0·84)	49 (16 to 69)	0·01
≤4 months after vaccination										
	Nepal	20	615·7	32·5	22	615·3	35·8	0·91 (0·50 to 1·66)	9 (−66 to 50)	0·76
	Mali	7	694·1	10·0	26	688·0	37·8	0·27 (0·12 to 0·61)	73 (39 to 88)	0·002
	South Africa	16	347·2	46·1	28	343·6	81·5	0·56 (0·30 to 1·04)	44 (−4 to 70)	0·07
	Pooled	43	1657·0	26·0	76	1647·0	46·1	0·56 (0·39 to 0·82)	44 (18 to 61)	0·003
≤6 months after vaccination										
	Nepal	26	910·2	28·6	30	909·7	33·0	0·87 (0·51 to 1·46)	13 (−46 to 49)	0·59
	Mali	9	1012·6	8·9	37	1004·9	36·8	0·24 (0·12 to 0·50)	76 (50 to 88)	<0·001
	South Africa	18	494·5	36·4	36	490·2	73·4	0·50 (0·28 to 0·87)	50 (13 to 72)	0·02
	Pooled	53	2417·2	21·9	103	2404·8	42·8	0·51 (0·37 to 0·71)	49 (29 to 63)	<0·001
**Non-cumulative episodes**										
≤2 months after vaccination										
	Nepal	9	307·8	29·2	12	307·7	39·0	0·75 (0·32 to 1·78)	25 (−78 to 68)	0·51
	Mali	6	350·1	17·1	19	346·5	54·8	0·31 (0·12 to 0·78)	69 (22 to 88)	0·01
	South Africa	8	175·8	45·5	14	173·5	80·7	0·56 (0·24 to 1·34)	44 (−34 to 76)	0·20
	Pooled	23	833·7	27·6	45	827·9	54·4	0·51 (0·31 to 0·84)	49 (16 to 69)	0·01
>2–4 months after vaccination										
	Nepal	11	307·8	35·7	10	307·6	32·5	1·10 (0·47 to 2·59)	−10 (−159 to 53)	0·83
	Mali	1	344·0	2·9	7	341·4	20·5	0·14 (0·02 to 1·15)	86 (−15 to 98)	0·07
	South Africa	8	171·4	46·6	14	170·1	82·3	0·57 (0·24 to 1·35)	43 (−35 to 76)	0·20
	Pooled	20	823·3	24·3	31	819·1	37·8	0·64 (0·36 to 1·12)	36 (−12 to 64)	0·12
>4–6 months after vaccination										
	Nepal	6	294·6	20·4	8	294·4	27·2	0·75 (0·26 to 2·16)	25 (−116 to 74)	0·59
	Mali	2	318·4	6·0	11	316·8	33·3	0·18 (0·04 to 0·82)	81 (18 to 96)	0·03
	South Africa	2	147·2	13·6	8	146·6	54·6	0·25 (0·05 to 1·17)	75 (−17 to 95)	0·08
	Pooled	10	760·2	13·2	27	757·8	35·6	0·37 (0·18 to 0·76)	63 (24 to 82)	0·01

Women might have had repeat episodes of influenza.

* Person-time was calculated consistently across sites and might be different from original site publication.

**Figure 2:**
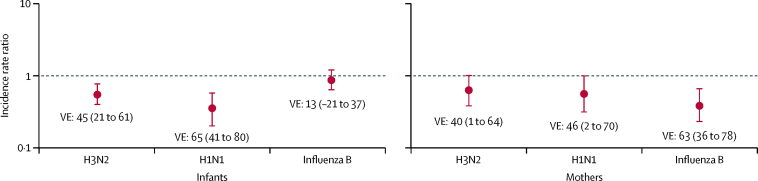
Incidence rate ratios and corresponding vaccine efficacies of maternal IIV on episodes of PCR-confirmed influenza in mothers and infants by strain

Among infants, pooled maternal vaccination was 65% (95% CI 41 to 80) efficacious against H1N1 influenza and 45% (21 to 61) efficacious against H3N2 (figure 2; appendix p 4). The vaccine was not efficacious in pooled analysis against influenza B in infants (vaccine efficacy 13%, 95% CI −21 to 37; figure 2; appendix p 4). For women, IIV had a pooled efficacy of 46% (95% CI 2 to 70) against H1N1 influenza, 40% (95% CI 1 to 64) against H3N2, and 63% (95% CI 36 to 78) against influenza B (figure 2; appendix p 5).

**Figure 3:**
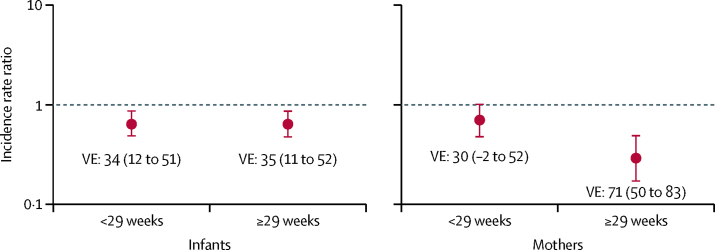
Incidence rate ratios and corresponding vaccine efficacies of maternal IIV on episodes of PCR-confirmed influenza in mothers and infants by gestational age at vaccination The cutoff of 29 weeks gestational age reflects the pooled median gestational age at vaccination.

In infants, maternal vaccination was similarly efficacious against PCR-confirmed influenza when vaccinated before 29 weeks gestational age (vaccine efficacy 34%, 95% CI 12 to 51; figure 3; table 4), and after 29 weeks gestational age (35%, 11 to 52). In women vaccinated at or after 29 weeks gestational age, maternal IIV was 71% (95% CI 50 to 83) efficacious for protecting against influenza (figure 3; table 4), whereas the estimate of vaccine efficacy was 30% (–2 to 52) in women vaccinated before 29 weeks gestational age.

**Table 4:** Efficacy of maternal inactivated influenza vaccine on episodes of PCR-confirmed influenza in infants and women by gestational age

		**Intervention**			**Control**			**Incidence rate ratio (95% CI)**	**Vaccine efficacy, % (95% CI)**	**p value**
		Cases	Person-time	Incidence, per 1000 person-years	Cases	Person-time	Incidence, per 1000 person-years			
**Infants**										
<29 weeks gestational age										
	Nepal	62	709·0	87·4	90	722·5	124·6	0·70 (0·51 to 0·97)	30 (3 to 49)	0·03
	Mali	8	135·0	59·2	16	135·4	118·2	0·50 (0·21 to 1·17)	50 (−17 to 79)	0·11
	South Africa	7	289·8	24·2	12	282·7	42·4	0·57 (0·22 to 1·44)	43 (−44 to 78)	0·24
	Pooled	77	1153·2	66·8	118	1156·3	102·0	0·66 (0·49 to 0·88)	34 (12 to 51)	0·004
≥29 weeks gestational age										
	Nepal	12	163·2	73·5	15	155·9	96·2	0·76 (0·36 to 1·63)	24 (−63 to 64)	0·49
	Mali	42	796·0	52·8	61	793·7	76·8	0·69 (0·46 to 1·02)	31 (−2 to 54)	0·06
	South Africa	12	178·8	67·1	25	184·1	135·8	0·49 (0·25 to 0·98)	51 (2 to 75)	0·04
	Pooled	66	1143·3	57·7	101	1139·4	88·6	0·65 (0·48 to 0·89)	35 (11 to 52)	0·01
**Mothers**										
<29 weeks gestational age										
	Nepal	27	1243·2	21·7	40	1246·2	32·1	0·68 (0·42 to 1·10)	32 (−10 to 58)	0·12
	Mali	4	207·6	19·3	5	206·6	24·2	0·80 (0·21 to 2·97)	20 (−197 to 79)	0·73
	South Africa	14	476·3	29·4	19	460·4	41·3	0·71 (0·36 to 1·42)	29 (−42 to 64)	0·34
	Pooled	45	1928·0	23·3	64	1914·2	33·4	0·70 (0·48 to 1·02)	30 (−2 to 52)	0·06
≥29 weeks gestational age										
	Nepal	4	227·1	17·6	4	218·1	18·3	0·96 (0·24 to 3·84)	4 (−284 to 76)	0·95
	Mali	8	1052·0	7·6	36	1047·2	34·4	0·22 (0·10 to 0·48)	78 (52 to 90)	<0·001
	South Africa	5	231·2	21·6	19	240·4	79·0	0·27 (0·10 to 0·73)	73 (27 to 90)	0·01
	Pooled	17	1510·3	11·2	59	1506·9	39·2	0·29 (0·17 to 0·50)	71 (50 to 83)	<0·001

Women and infants could have had repeat episodes of influenza.

Although maternal influenza immunisation was protective against low birthweight (RR 0·85, 95% CI 0·74–0·96) in Nepal; overall there was no association between maternal immunisation and low birthweight (0·96, 0·87–1·06), stillbirth (1·02, 0·74–1·42), preterm birth (0·97, 0·87–1·08), and small for gestational age birth (0·99, 0·93–1·06; table 5).

**Table 5:** Effect of maternal inactivated influenza vaccine on birth outcomes

	**Intervention**			**Control**			**Risk ratio (95% CI)**	**p value**
	Cases	Infants, n	Incidence, per 100 infants	Cases	Infants, n	Incidence, per 100 infants		
**Low birthweight**								
Nepal	318	1386	22·9	370	1367	27·1	0·85 (0·74–0·96)	0·01
Mali	191	2061	9·3	165	2038	8·1	1·14 (0·94–1·40)	0·18
South Africa	133	1024	13·0	122	1021	11·9	1·09 (0·86–1·37)	0·48
Pooled	642	4471	14·4	657	4426	14·8	0·96 (0·87–1·06)	0·39
**Stillbirth**								
Nepal	33	1858	1·8	31	1861	1·7	1·07 (0·66–1·73)	0·80
Mali	24	2088	1·1	30	2071	1·4	0·79 (0·46–1·35)	0·40
South Africa	15	1044	1·4	9	1037	0·9	1·66 (0·73–3·77)	0·23
Pooled	72	4985	1·4	70	4965	1·4	1·02 (0·74–1·42)	0·88
**Preterm birth**								
Nepal	234	1767	13·2	255	1760	14·5	0·91 (0·78–1·08)	0·28
Mali	201	2064	9·7	204	2041	10·0	0·97 (0·81–1·17)	0·78
South Africa	108	1026	10·5	96	1023	9·4	1·12 (0·86–1·46)	0·39
Pooled	543	4857	11·2	555	4824	11·5	0·97 (0·87–1·08)	0·60
**Small for gestational age**								
Nepal	553	1307	42·3	574	1301	44·1	0·96 (0·88–1·05)	0·35
Mali	435	1685	25·8	420	1640	25·8	1·01 (0·90–1·13)	0·89
South Africa	117	715	16·4	102	740	13·8	1·19 (0·93–1·52)	0·17
Pooled	1105	3707	29·8	1096	3681	29·8	0·99 (0·93–1·06)	0·80

In pooled analyses of growth outcomes at 6 months of age, the intervention and control groups were similar in terms of severely underweight (RR 0·88, 95% CI 0·66–1·16), severe wasting (0·91, 0·70–1·19), and severe stunting (0·86, 0·68–1·10; table 6). Similarly, underweight (0·99, 0·87–1·12), wasting (1·02, 0·89–1·18), and stunting (0·93, 0·83–1·05) were not substantively different between the intervention and control groups (table 6). In pooled analyses stratified by preterm, small for gestational age, and low birthweight status, there was no association between the intervention and control groups in terms of anthropometric measures (appendix pp 6–8). Median centile change from birth to 6 months of age was similar between the IIV and control groups for both weight and length, although the median centile change in weight was greatest in the vaccinated group in South Africa (appendix p 9). There was no difference in mean weight or length at birth or 6 months between study groups (appendix p 10).

**Table 6:** Effect of maternal inactivated influenza vaccine on 6-month-old infant weight-for-age, weight-for-length, and length-for-age

	**Intervention**		**Control**		**Risk ratio (95% CI)**	**p value**
	Infants, n	Incidence, per 1000 infants	Infants, n	Incidence, per 1000 infants		
**Underweight**						
Nepal	233	182·6	228	182·4	1·00 (0·85–1·18)	0·99
Mali	124	80·7	137	90·3	0·89 (0·71–1·13)	0·34
South Africa	49	66·8	40	54·6	1·22 (0·82–1·83)	0·33
Pooled	406	114·5	405	115·7	0·99 (0·87–1·12)	0·83
**Severely underweight**						
Nepal	55	43·1	69	55·2	0·78 (0·55–1·10)	0·16
Mali	26	16·9	25	16·5	1·03 (0·60–1·77)	0·92
South Africa	11	15·0	9	12·3	1·22 (0·51–2·93)	0·66
Pooled	92	26·0	103	29·4	0·88 (0·66–1·16)	0·35
**Wasting**						
Nepal	140	109·7	129	103·2	1·06 (0·85–1·33)	0·60
Mali	167	108·7	172	113·4	0·96 (0·78–1·17)	0·68
South Africa	58	79·1	50	68·3	1·16 (0·80–1·67)	0·43
Pooled	365	103·0	351	100·3	1·02 (0·89–1·18)	0·72
**Severe wasting**						
Nepal	33	25·9	47	37·6	0·69 (0·44–1·07)	0·09
Mali	48	31·2	45	29·7	1·05 (0·70–1·57)	0·80
South Africa	21	28·6	18	24·6	1·16 (0·62–2·17)	0·63
Pooled	102	28·8	110	31·4	0·91 (0·70–1·19)	0·51
**Stunting**						
Nepal	174	136·4	185	148·0	0·92 (0·76–1·12)	0·40
Mali	99	64·4	77	50·8	1·27 (0·95–1·70)	0·11
South Africa	170	23·2	206	28·1	0·82 (0·69–0·98)	0·03
Pooled	443	125·0	468	133·8	0·93 (0·83–1·05)	0·26
**Severe stunting**						
Nepal	26	20·4	40	32·0	0·64 (0·39–1·04)	0·07
Mali	20	13·0	16	10·5	1·23 (0·64–2·37)	0·53
South Africa	70	95·5	78	106·6	0·90 (0·66–1·22)	0·48
Pooled	116	32·7	134	38·3	0·86 (0·68–1·10)	0·22

Most two-stage meta-analyses done using site risk ratios showed low to moderate heterogeneity across sites. Exceptions included maternal PCR-confirmed influenza 0–6 months after vaccination and efficacy among women against H1N1, which had high heterogeneity (appendix p 11).

## Discussion

In this analysis, we found that IIV administered in pregnancy was associated with an overall efficacy of 35% for PCR-confirmed influenza among infants younger than 6 months of age. The protection against infant influenza was greater in the first 2 months of life, with 56% efficacy, but the vaccine did not show efficacy after 4 months of age. Previous reports of the individual RCTs documented heterogonous estimates of maternal IIV efficacy in infants, with estimates ranged from 63% in Bangladesh to 30% in Nepal. Such heterogeneity in efficacy estimates is unsurprising because the effects of influenza vaccination are known to vary by time and location. An overall 35% efficacy and 56% efficacy among infants younger than 2 months could, however, have substantial public health implications, because young infants tend to have more severe disease compared with older children.^1^

The mechanism behind a woman's immune response to immunisation by gestational age and the optimal time to administer maternal influenza immunisation is unclear. We found lower vaccine efficacy in women who were vaccinated earlier than the median gestational age at vaccination (29 weeks) than those vaccinated later. One explanation for this finding might be that the assessment of efficacy was underpowered for women vaccinated before 29 weeks gestational age, because the point estimate suggests that there might be efficacy despite wide CIs. There was also lower disease burden in women compared with infants, which might explain why we were able to see an effect among infants whose mothers were vaccinated during this period, but were not able to see an effect among the women. Women with previous influenza infection were not excluded from this analysis. Due to the general misunderstanding of what is truly influenza as opposed to influenza-like illness, it is unlikely that women without previous influenza infection were self-selected into the trials. Differences in when sites enrolled and vaccinated women could be another explanation for the lower efficacy in women vaccinated earlier. In Mali, where the greatest efficacy of the three sites was shown, women were vaccinated later in pregnancy compared with Nepal and South Africa. Previous studies have shown that antibody response to influenza immunisation might decline as pregnancy progresses, further suggesting that our findings might be due to an issue of power rather than a difference in efficacy.^20^

With regard to infant protection, evidence suggests that transplacental transfer of antibodies might be less efficient the closer vaccination is to delivery, although we found vaccine efficacy to be similar among infants whose mothers received the influenza vaccination before and after 29 weeks gestation.^21^, ^22^ Owing to the possibility of losing power in this analysis and the differences in timing of vaccination between sites, our results should be interpreted with caution. Additional data will be useful in developing recommendations on the optimal immunisation timing for maternal and infant protection.

According to this most comprehensive analysis to date of vaccine efficacy—based on RCTs of maternal influenza vaccination—IIV was efficacious in protecting women throughout the duration of the study, although further analysis of the South Africa trial showed that women vaccinated in 2011 were still protected during the 2012 influenza season.^23^ For infants, protection lasted only in the first 4 months of life. This is in line with the persistence of antibodies in infants found in Mali, South Africa, and Bangladesh trials, which declined over the first 6 months of life.^9^, ^10^, ^24^ Consistent with our findings, the resulting infant protection against PCR-confirmed influenza lasted for the first 8 weeks of life in South Africa, and declined as follow-up extended from 4 months to 6 months of age in Mali.^9^, ^10^ In Bangladesh, protection against respiratory illness with fever until 5–6 months of age was reported.^4^ These findings have policy implications. No influenza vaccine is licensed for infants younger than 6 months of age. Therefore, even with a robust maternal influenza vaccination programme, an infant immunity gap of at least 2 months could remain. Strategies to overcome this immunity gap could include studies of high dose or adjuvanted vaccines and evaluation of infant influenza immunisation starting at 4 months. It might be worth investigating effects in women and infants if more immunogenic vaccines are developed in the future.

In addition to protection of infants and women against influenza through maternal immunisation, evidence from RCTs suggests protection against other outcomes. For example, in a previous pooled analysis of the same trials,^25^ we reported that maternal IIV was 20% efficacious in protecting young infants against all-cause severe pneumonia. The protection was greatest during periods of high influenza circulation, indicating that maternal IIV could be particularly useful during pandemics.^25^ Similarly, a post-hoc analysis of the South Africa trial reported a reduction of around 50% in PCR-confirmed pertussis in influenza-vaccinated women.^26^ Previously, data from the Bangladesh maternal influenza immunisation trial indicated that maternal IIV enhanced the effect of the 7-valent pneumococcal conjugate vaccine on outcomes analogous to influenza-like illness and medically attended respiratory illness; whereas, on their own, both the pneumococcal vaccine and IIV had minimal or no effect on these outcomes.^27^

The efficacy estimates in this paper are based on primary mild illness and on confirmation of influenza infection by PCR, and might underestimate the total effect of maternal vaccination based on the efficacy against serological confirmed illness.^28^ These efficacy estimates could have a bearing on prevention of secondary complications, such as secondary bacterial infection, which likely form the basis for protection against all-cause pneumonia.

We did not find an overall association between influenza immunisation in pregnancy and low birthweight, stillbirth, preterm birth, small for gestational age, and infant growth in the first 6 months of life. In the site-specific analyses, consistent with previous reports, there was an association between maternal influenza immunisation and low birthweight in Nepal during the two cohorts of the trial combined. However, there was no association between maternal influenza immunisation and birth outcomes in Mali and South Africa. In the earlier Bangladesh trial of maternal IIV, there was a difference in mean birthweight between the IIV and control groups.^4^

There could be multiple reasons for the heterogeneity in low birthweight estimates by site. For example, IIV was administered at earlier gestational ages in Nepal compared with Mali and South Africa. Moreover, in Nepal, there was year-round circulation of influenza virus, whereas in Mali and South Africa, the circulation was more concentrated to a part of the study period. Early vaccination and year-round circulation in Nepal could have resulted in a greater opportunity to protect against adverse effects of influenza on fetal growth, and women in Nepal were exposed to influenza longer during pregnancy than women in Mali or South Africa. Nepal and Mali also used a year-round enrolment strategy, as opposed to enrolling women to correspond with the influenza season, as was done in South Africa.^8^, ^9^, ^12^ Notably, the mean birthweight in the control groups was substantially heavier in Mali and South Africa compared with Nepal. This is true not only for the trial participants, but also for the populations of the areas where these trials were done. The Nepal trial was the only one with low birthweight as the a-priori co-primary endpoint, with the trial powered to detect a protective effect of IIV on infant birthweight, although this was true for the two cohorts of the trial combined and not for each cohort individually.^12^

Given the heterogeneity in findings, the interpretation of evidence from the maternal IIV trials regarding adverse birth outcomes is nuanced. The vaccine might be protective for birthweight in south Asian populations or in populations with a greater prevalence of maternal malnutrition and lower baseline birthweight, and there might not be a global effect. The association between maternal anthropometry and maternal influenza immunisation could be a worthwhile area for future research to investigate. However, the results from our pooled analysis suggest that the available evidence does not warrant inclusion of most birth outcome data in global impact models for decision making regarding maternal influenza vaccination.

A goal of all RCTs is to assess the safety of the intervention evaluated in the trial. Our results provide further evidence in support of the safety of maternal influenza vaccines. However, the vaccines in these trials were administered primarily during the third trimester—although in Nepal the gestational age at vaccination ranged from 17 to 34 weeks—which would not capture early stillbirths adequately. Therefore, care should be exercised in generalising findings to vaccination outside the gestational period covered by this study.

Our analysis has some limitations. For example, although the three trial teams coordinated their study procedures, there were some differences between their protocols. These differences (and similarities) have been previously described in detail.^6^ One difference of note is that the sites used different methods to estimate gestational age. However, the overall consistency in findings across sites suggests our estimates are robust. Additionally, although the combined cohort size of 10 002 women and 9800 eligible infants provided sufficient power for many analyses, this sample size is not sufficient for rare outcomes such as stillbirth, and might affect the findings in some of the analyses done in specific time frames after vaccination or by gestational age at vaccination. Therefore, our trial-based findings will have to be complemented by (ideally prospective) cohort studies. Moreover, although inclusion of RCTs done at three different locations makes the findings more generalisable, influenza epidemiology varies substantially by location and time, as does underlying population nutritional status. Therefore, pooled estimates from this analysis should be interpreted accordingly.

Our findings lend support to global and several national recommendations to vaccinate pregnant women against influenza. However, the findings also suggest that infant protection from maternal influenza immunisation was limited to 4 months of age. The estimates of efficacy, and evidence of impact against PCR-confirmed influenza and safety in terms of adverse birth outcomes, should be incorporated into any further consideration of maternal influenza immunisation recommendations.

## Data sharing

We are committed to open data sharing and knowledge. However, our trials were done in three different countries with various legal standards. We will therefore evaluate requests for data sharing on a case-by-case basis. Requests for data can be made by contacting the corresponding author (SBO).
