# Supplementary material for: Efficacy, duration of protection, birth outcomes, and infant growth associated with influenza vaccination in pregnancy: a pooled analysis of three randomised controlled trials
Source: Lancet Respir Med. 2020 Jun;8(6):597–608. doi: 10.1016/S2213-2600(19)30479-5 (PMC7284303; doi:10.1016/S2213-2600(19)30479-5)
Supplement: Supplementary appendix [file mmc1.pdf]

THE LANCET
Respiratory Medicine

Supplementary appendix

This appendix formed part of the original submission and has been peer reviewed. We post it as supplied by the authors.

Supplement to: Omer SB, Clark DR, Madhi SA, et al, for the BMGF Supported Maternal Influenza Immunization Trials Investigators Group. Efficacy, duration of protection, birth outcomes, and infant growth associated with influenza vaccination in pregnancy: a pooled analysis of three randomised controlled trials. *Lancet Respir Med* 2020; **8:** 597–608.

SUPPLEMENTAL MATERIALS

BMGF Supported Maternal Influenza Immunization Trials Investigators Group
Ordered by site and alphabetically.

Mali: William Blackwelder, PhD; Joseph Bresee, MD; Flanon Coulibaly, MD; Boubacar Diallo; Fatoumata Diallo, MD; Wilbur Chen, MD, MS; Moussa Doumbia, MD; Fadima Cheick Haidara, MD; Adama Mamby Keita, MD, Alexander Klimov, PhD; Mamoudou Kodio, PharmD; Karen Kotloff, MD; Myron M. Levine, MD, DTPH; Vladimir Mishcherkin MS; Uma Onwuchekwa; Sandra Panchalingam, PhD; Marcela Pasetti, PhD; Doh Sanogo, MD; Samba Sow, MD MS; Milagritos Tapia, MD; Boubou Tamboura, PharmD; Ibrahim Teguete, MD; Sharon Tennant, PhD, Awa Traore, PharmD; and John Treanor, MD.

Nepal: Janet A. Englund, MD; Joanne Katz, ScD; Subarna K. Khatry, MD; Jane Kuypers, PhD; Steven C. LeClerq, MPH; Luke C. Mullany, PhD; Laxman Shrestha, MD; Mark C. Steinhoff, MD; and James M. Tielsch, PhD.

South Africa: Peter V. Adrian, PhD; Clare L. Cutland, MD; Andrea Hugo, MD; Stephanie Jones, MD; Locadiah Kuwanda, MSc; Keith P. Klugman, MD, PhD; Shabir A. Madhi, MD, PhD; Kathleen M. Neuzil, MD; Nadia van Niekerk, BTech; Marta C. Nunes, PhD; Justin R. Ortiz, MD; Eric A.F. Simões, MD; Florette Treurnicht, PhD; Marietjie Venter, PhD; Avy Violari, MD; and Adriana Weinberg, MD.

Table S1. Efficacy of maternal IIV on first episodes of PCR-confirmed influenza in infants born to women vaccinated at any time pre-partum

	Intervention			Control					
	Cases	No. Infants	Incidence[1]	Cases	No. Infants	Incidence[1]	Risk Ratio (95% Confidence Interval)	Vaccine Efficacy % (95% Confidence Interval)	P-Value
**≤ 2 months of age**									
Nepal	20	1820	11.0	30	1826	16.4	0.67 (0.38, 1.17)	33 (-17, 62)	0.2
Mali	2	2064	1.0	10	2041	4.9	0.20 (0.04, 0.90)	80 (10, 96)	0.02
South Africa	2	1026	1.9	13	1023	12.7	0.15 (0.03, 0.68)	85 (32, 97)	0.004
Pooled	24	4910	4.9	53	4890	10.8	0.45 (0.28, 0.73)	55 (27, 72)	0.001
**≤ 4 months of age**									
Nepal	42	1820	23.1	60	1826	32.8	0.70 (0.48, 1.04)	30 (-4, 52)	0.09
Mali	11	2064	5.3	33	2041	16.2	0.33 (0.17, 0.65)	67 (35, 83)	<0.001
South Africa	14	1026	13.6	30	1023	29.3	0.46 (0.25, 0.87)	54 (13, 75)	0.01
Pooled	67	4910	13.6	123	4890	25.2	0.54 (0.40, 0.73)	46 (27, 60)	<0.0001
**≤ 6 months of age**									
Nepal	72	1820	39.6	101	1826	55.3	0.72 (0.53, 0.96)	28 (4, 47)	0.03
Mali	50	2064	24.2	75	2041	36.7	0.66 (0.46 0.94)	34 (6, 54)	0.02
South Africa	19	1026	18.5	37	1023	36.1	0.51 (0.30, 0.88)	49 (12, 70)	0.01
Pooled	141	4910	28.7	213	4890	43.6	0.66 (0.54, 0.81)	34 (19, 46)	<0.0001

[1] Per 1,000 infants

Table S2. Efficacy of maternal IIV on first episodes of PCR-confirmed influenza in women vaccinated at any time pre-partum

	Intervention			Control					
	Cases	No. Women	Incidence[1]	Cases	No. Women	Incidence[1]	Risk Ratio (95% Confidence Interval)	Vaccine Efficacy % (95% Confidence Interval)	P-Value
**During Pregnancy[2]**									
Nepal	19	1847	10.3	23	1846	12.4	0.82 (0.45, 1.51)	18 (-51, 55)	0.54
Mali	6	2108	2.8	17	2085	8.2	0.35 (0.14, 0.88)	65 (12, 86)	0.02
South Africa	11	1062	10.4	19	1054	18.0	0.57 (0.27, 1.20)	43 (-20, 73)	0.14
Pooled	36	5017	7.2	59	4985	11.8	0.61 (0.40, 0.92)	39 (8, 60)	0.02
**After Pregnancy[2]**									
Nepal	12	1847	6.5	19	1846	10.3	0.63 (0.31, 1.30)	27 (-30, 69)	0.14
Mali	7	2108	3.3	23	2085	11.0	0.30 (0.13, 0.70)	70 (30, 87)	0.003
South Africa	6	1062	5.6	19	1054	18	0.31 (0.12, 0.78)	67 (17, 87)	0.01
Pooled	25	5017	5.0	61	4985	12.2	0.41 (0.26, 0.65)	59 (35, 74)	<0.0001
**During Full Study Period**									
Nepal	31	1847	16.8	42	1846	22.8	0.74 (0.46, 1.17)	26 (-17, 54)	0.2
Mali	12	2108	5.7	40	2085	19.2	0.30 (0.16, 0.56)	70 (44, 84)	<0.0001
South Africa	19	1062	17.9	38	1054	36.0	0.50 (0.29, 0.86)	49 (12, 70)	0.01
Pooled	62	5017	12.4	120	4985	24.1	0.51 (0.38, 0.70)	48 (30, 42)	<0.0001

[1] Per 1,000 women

[2] Women missing a date of delivery were excluded from the during/after analyses

Table S3. Efficacy of maternal IIV on episodes of PCR-confirmed influenza in infants born to women vaccinated at any time pre-partum by strain

	Intervention			Control					
	Cases	Person-time	Incidence[1]	Cases	Person-time	Incidence[1]	Incidence Rate Ratio (95% Confidence Interval)	Vaccine Efficacy % (95% Confidence Interval)	P-Value
**H3N2**									
Nepal	18	896.9	20.1	35	899.9	38.9	0.52 (0.29, 0.91)	48 (9, 71)	0.02
Mali	22	931.0	10.6	36	929.0	17.6	0.61 (0.36, 1.04)	39 (-4, 64)	0.07
South Africa	7	468.5	14.9	14	466.8	30.0	0.50 (0.20, 1.23)	50 (-23, 80)	0.13
Pooled	47	2296.4	20.5	85	2295.7	37.0	0.55 (0.39, 0.79)	45 (21, 61)	0.001
**H1N1**									
Nepal	6	896.9	6.7	23	899.9	25.6	0.26 (0.11, 0.64)	64 (22, 83)	0.003
Mali	12	931.0	12.9	28	929.0	30.1	0.43 (0.22, 0.84)	57 (16, 78)	0.01
South Africa	0	468.5	0.0	1	466.8	2.1	--	--	--
Pooled	18	2296.4	7.8	52	2295.7	22.6	0.35 (0.20, 0.59)	65 (41, 80)	0.001
**B**									
Nepal	42	896.9	46.8	42	899.9	46.7	1.00 (0.65, 1.54)	0 (-54, 35)	0.99
Mali	14	931.0	15.0	13	929.0	14.0	1.07 (0.50, 2.29)	-7 (-129, 50)	0.85
South Africa	12	468.5	25.6	23	466.8	49.3	0.52 (0.26, 1.04)	48 (-4, 74)	0.06
Pooled	68	2296.4	29.6	78	2295.7	34.0	0.87 (0.63, 1.21)	13 (-21, 37)	0.41

[1] Per 1,000 infant-years

Table S4. Efficacy of maternal IIV on episodes of PCR-confirmed influenza in pregnant women vaccinated at any time pre-partum by strain

	Intervention			Control					
	Cases	Person-time	Incidence[1]	Cases	Person-time	Incidence[1]	Incidence Rate Ratio (95% Confidence Interval)	Vaccine Efficacy % (95% Confidence Interval)	P-Value
**H3N2**									
Nepal	9	1471.3	6.1	11	1466.4	7.5	0.82 (0.34, 1.97)	18 (-97, 46)	0.65
Mali	5	1259.6	4.0	7	1253.8	5.6	0.71 (0.22, 2.24)	29 (-124, 78)	0.56
South Africa	10	707.4	14.1	22	700.8	31.4	0.45 (0.21, 0.95)	55 (5, 79)	0.04
Pooled	24	2417.3	9.9	40	2404.4	16.6	0.60 (0.36, 0.99)	40 (1, 64)	0.04
**H1N1**									
Nepal	9	1471.3	6.1	6	1466.4	4.1	1.50 (0.53, 4.20)	-50 (-320, 47)	0.44
Mali	3	1259.6	2.4	19	1253.8	15.2	0.16 (0.05, 0.53)	84 (47, 95)	0.003
South Africa	5	707.4	7.1	6	700.8	8.6	0.82 (0.25, 2.70)	18 (-170, 75)	0.75
Pooled	17	2417.3	7	31	2404.4	12.9	0.54 (0.30, 0.98)	46 (2, 70)	0.04
**B**									
Nepal	10	1471.3	6.8	23	1466.4	15.7	0.43 (0.21, 0.91)	57 (9, 79)	0.03
Mali	4	1259.6	3.2	15	1253.8	12.0	0.26 (0.09, 0.80)	74 (20, 91)	0.02
South Africa	4	707.4	5.6	10	700.8	14.3	0.40 (0.12, 1.26)	60 (-26, 88)	0.12
Pooled	18	2417.3	7.4	48	2404.4	20.0	0.37 (0.22, 0.64)	63 (36, 78)	<0.0001 1

[1] Per 1,000 person-years

Table S5. Pooled impact of maternal IIV on 6-month old infant **weight-for-age** by LBW, preterm birth, and SGA

	Intervention		Control					Intervention		Control				
	No. Infants	Incidence[1]	No. Infants	Incidence[1]	RR	95% CI	P-Value	No. Infants	Incidence[1]	No. Infants	Incidence[1]	RR	95% CI	P-Value
	**Non-LBW**							**LBW**						
Underweight	248	81.7	254	84.8	0.96	(0.82, 1.14)	0.67	149	309.8	146	308.7	1.00	(0.83, 1.21)	0.97
Severely Underweight	46	15.2	53	17.7	0.86	(0.58, 1.27)	0.44	45	93.6	49	103.6	0.90	(0.61, 1.33)	0.60
	**Term**							**Preterm**						
Underweight	329	104.0	321	102.6	1.01	(0.88, 1.17)	0.86	77	202.1	83	223.7	0.90	(0.68, 1.19)	0.47
Severely Underweight	62	19.6	74	23.7	0.83	(0.59, 1.16)	0.27	30	78.7	28	75.5	1.04	(0.64, 1.71)	0.87
	**AGA**							**SGA**						
Underweight	136	69.7	142	73.1	0.95	(0.76, 1.20)	0.68	172	214.7	173	221.8	0.97	(0.80, 1.17)	0.73
Severely Underweight	26	13.3	23	11.8	1.12	(0.64, 1.97)	0.68	47	58.7	54	69.2	0.85	(0.58, 1.24)	0.39

[1] Per 1,000 infants

Table S6. Pooled impact of maternal IIV on 6-month old infant **weight-for-length** by LBW, preterm birth, and SGA

	Intervention		Control					Intervention		Control				
	No. Infants	Incidence[1]	No. Infants	Incidence[1]	RR	95% CI	P-Value	No. Infants	Incidence[1]	No. Infants	Incidence[1]	RR	95% CI	P-Value
	**Non-LBW**							**LBW**						
Wasting	279	92.0	274	91.5	1.01	(0.86, 1.18)	0.95	80	166.3	74	156.4	1.06	(0.80, 1.42)	0.68
Severe Wasting	78	25.7	86	28.7	0.90	(0.66, 1.21)	0.47	24	49.9	23	48.6	1.03	(0.59, 1.79)	0.93
	**Term**							**Preterm**						
Wasting	306	96.7	302	96.6	1.00	(0.86, 1.16)	0.99	59	154.8	48	129.4	1.20	(0.84, 1.70)	0.32
Severe Wasting	89	28.1	93	29.7	0.94	(0.71, 1.26)	0.70	13	34.1	17	45.8	0.74	(0.37, 1.51)	0.42
	**AGA**							**SGA**						
Wasting	171	87.6	156	80.3	1.09	(0.89, 1.34)	0.41	122	152.3	119	152.6	1.00	(0.79, 1.26)	0.99
Severe Wasting	45	23.1	45	23.2	1.00	(0.66, 1.50)	0.98	42	52.4	41	52.6	1.00	(0.66, 1.52)	0.99

[1] Per 1,000 infants

Table S7. Pooled impact of maternal IIV on 6-month old infant **length-for-age** by preterm birth, SGA and LBW

	Intervention		Control					Intervention		Control				
	No. Infants	Incidence[1]	No. Infants	Incidence[1]	RR	95% CI	P-Value	No. Infants	Incidence[1]	No. Infants	Incidence[1]	RR	95% CI	P-Value
	**Non-LBW**							**LBW**						
Stunting	299	98.5	326	108.8	0.91	(0.78, 1.05)	0.19	139	289.0	136	287.5	1.01	(0.82, 1.23)	0.96
Severe Stunting	70	23.1	85	28.4	0.81	(0.59, 1.11)	0.19	46	95.6	46	97.3	0.98	(0.67, 1.45)	0.93
	**Term**							**Preterm**						
Stunting	362	114.4	385	123.1	0.93	(0.81, 1.06)	0.29	81	212.6	82	221.0	0.96	(0.73, 1.26)	0.78
Severe Stunting	85	26.9	108	34.5	0.78	(0.59, 1.03)	0.08	31	81.4	26	70.1	1.16	(0.70, 1.92)	0.56
	**AGA**							**SGA**						
Stunting	187	95.8	219	112.7	0.85	(0.71, 1.02)	0.09	137	171.0	130	166.7	1.03	(0.82, 1.28)	0.82
Severe Stunting	47	24.1	54	27.8	0.87	(0.59, 1.28)	0.47	32	40.0	34	43.6	0.92	(0.57, 1.47)	0.72

[1] Per 1,000 infant-years

Table S8. Median infant centile change from birth to 6 months

	Intervention		Control		
	Median Change	(IQR)	Median Change	(IQR)	P-Value[1]
**Weight**					
Nepal	0.2	(-13.8, 15.5)	0.8	(-11.8, 17.9)	0.18
Mali	2.7	(-16.0, 25.2)	3.4	(-14.5, 26.6)	0.15
South Africa	11.8	(-3.6, 33.2)	8.7	(-10.5, 32.2)	0.05
Pooled	2.7	(-13.3, 23.8)	3.1	(-12.8, 24.3)	0.44
**Length**					
Nepal	-1.8	(-20.4, 12.0)	-1.0	(-19.9, 11.2)	0.57
Mali	-0.6	(-24.3, 22.0)	0	(-24.6, 20.8)	0.94
South Africa	-12.5	(-50.8, 12.2)	-15.9	(-57.9, 11.8)	0.12
Pooled	-3.8	(-30.1, 14.9)	-4.0	(-30.3, 13.5)	0.71

1 Intervention and control compared using Wilcoxon Rank Sum Test

Table S9. Mean weight (kg) and length (cm) of infants by age and site

	Weight (SD)			Length (SD)		
	Intervention	Control	P-Value[1]	Intervention	Control	P-Value[1]
**Birth**						
Nepal	2.90 (0.55)	2.88 (0.56)	0.30	48.29 (2.52)	48.22 (2.56)	0.51
Mali	3.02 (0.47)	3.02 (0.44)	0.86	49.49 (1.91)	49.54 (1.80)	0.37
South Africa	3.06 (0.48)	3.08 (0.48)	0.27	49.78 (3.44)	49.97 (3.41)	0.21
Pooled	2.31 (1.32)	2.30 (1.31)	0.87	49.20 (2.58)	49.25 (2.56)	0.38
**6 months**						
Nepal	6.80 (0.97)	6.79 (1.02)	0.75	64.39 (2.39)	64.33 (2.54)	0.53
Mali	7.30 (0.97)	7.32 (0.98)	0.58	66.60 (2.63)	66.63 (2.53)	0.76
South Africa	7.54 (1.12)	7.52 (1.07)	0.71	65.07 (4.22)	64.89 (4.49)	0.44
Pooled	7.17 (1.04)	7.17 (1.05)	0.95	65.49 (3.12)	65.44 (3.22)	0.56

[1] Intervention and control compared using two-sample t-test

Table S10. I-squared test of heterogeneity based on meta-analysis of risk ratios across sites

Time Frame	Strain	Gestational Age at Vaccination	I^2 (%)
**Infants**			
≤ 2 Months of Age	All	All	60.1
≤ 4 Months of Age	All	All	49.4
≤ 6 Months of Age	All	All	0
> 2, ≤ 4 Months of Age	All	All	0
> 4, ≤ 6 Months of Age	All	All	0
≤ 6 Months of Age	H3N2	All	0
≤ 6 Months of Age	H1N1	All	0
≤ 6 Months of Age	Flu B	All	27.0
≤ 6 Months of Age	All	<29 weeks	0
≤ 6 Months of Age	All	≥29 weeks	0
LBW	-	All	62.6
Stillbirth	-	All	7.6
Preterm birth	-	All	0
SGA	-	All	0
Underweight	-	All	0
Severely underweight	-	All	0
Wasting	-	All	0
Severe wasting	-	All	20.8
Stunting	-	All	58.4
Severe stunting	-	All	23.4
**Women**			
During Pregnancy	All	All	15.2
After Pregnancy	All	All	8.9
Full Study	All	All	60.8
≤ 2 Months After Vaccination	All	All	0
≤ 4 Months After Vaccination	All	All	63.5
≤ 6 Months After Vaccination	All	All	76.2
> 2, ≤ 4 Months After Vaccination	All	All	50.6
> 4, ≤ 6 Months After Vaccination	All	All	40.4
Full Study	H3N2	All	0
Full Study	H1N1	All	75.5
Full Study	Flu B	All	0
Full Study	All	<29 weeks	0
Full Study	All	≥29 weeks	39.2
